# Pediatric reporting of genomic results study (PROGRESS): a mixed-methods, longitudinal, observational cohort study protocol to explore disclosure of actionable adult- and pediatric-onset genomic variants to minors and their parents

**DOI:** 10.1186/s12887-020-02070-4

**Published:** 2020-05-15

**Authors:** Juliann M. Savatt, Jennifer K. Wagner, Steven Joffe, Alanna Kulchak Rahm, Marc S. Williams, Angela R. Bradbury, F. Daniel Davis, Julie Hergenrather, Yirui Hu, Melissa A. Kelly, H. Lester Kirchner, Michelle N. Meyer, Jessica Mozersky, Sean M. O’Dell, Josie Pervola, Andrea Seeley, Amy C. Sturm, Adam H. Buchanan

**Affiliations:** 1 Genomic Medicine Institute, Geisinger, Danville, PA USA; 2 Center for Translational Bioethics and Health Care Policy, Geisinger, Danville, PA USA; 3 Department of Medical Ethics and Health Policy, University of Pennsylvania Perelman School of Medicine, Philadelphia, PA USA; 4 Department of Medicine, Division of Hematology-Oncology, University of Pennsylvania, Philadelphia, PA USA; 5 Department of Medical Ethics and Health Policy, University of Pennsylvania, Philadelphia, PA USA; 6 Department of Psychiatry and Behavioral Health, Geisinger, Danville, PA USA; 7 Department of Population Health Sciences, Geisinger, Danville, PA USA; 8 Bioethics Research Center, Washington University School of Medicine in St. Louis, St. Louis, MO USA; 9 Department of Pediatrics, Geisinger, Danville, PA USA

**Keywords:** Return of genomic results, Genomic medicine, Secondary findings, Pediatrics, *BRCA1*, *BRCA2*, Lynch syndrome

## Abstract

**Background:**

Exome and genome sequencing are routinely used in clinical care and research. These technologies allow for the detection of pathogenic/likely pathogenic variants in clinically actionable genes. However, fueled in part by a lack of empirical evidence, controversy surrounds the provision of genetic results for adult-onset conditions to minors and their parents. We have designed a mixed-methods, longitudinal cohort study to collect empirical evidence to advance this debate.

**Methods:**

Pediatric participants in the Geisinger MyCode® Community Health Initiative with available exome sequence data will have their variant files assessed for pathogenic/likely pathogenic variants in 60 genes designated as actionable by MyCode. Eight of these genes are associated with adult-onset conditions (Hereditary Breast and Ovarian Cancer Syndrome (HBOC), Lynch syndrome, *MUTYH*-associated polyposis, *HFE-*Associated Hereditary Hemochromatosis), while the remaining genes have pediatric onset. Prior to clinical confirmation of results, pediatric MyCode participants and their parents/legal guardians will be categorized into three study groups: 1) those with an apparent pathogenic/likely pathogenic variant in a gene associated with adult-onset disease, 2) those with an apparent pathogenic/likely pathogenic variant in a gene associated with pediatric-onset disease or with risk reduction interventions that begin in childhood, and 3) those with no apparent genomic result who are sex- and age-matched to Groups 1 and 2. Validated and published quantitative measures, semi-structured interviews, and a review of electronic health record data conducted over a 12-month period following disclosure of results will allow for comparison of psychosocial and behavioral outcomes among parents of minors (ages 0–17) and adolescents (ages 11–17) in each group.

**Discussion:**

These data will provide guidance about the risks and benefits of informing minors and their family members about clinically actionable, adult-onset genetic conditions and, in turn, help to ensure these patients receive care that promotes physical and psychosocial health.

**Trial registration:**

ClinicalTrials.gov Identifier: NCT03832985. Registered 6 February 2019

## Background

Exome and genome sequencing are increasingly integrated into clinical care and research [1–7], providing an opportunity to examine sequence data for pathogenic/likely pathogenic variants in clinically actionable genes. However, the potential benefits and harms of returning genetic results to minors (ages 0–17) and their parents/legal guardians (hereafter referred to as “parents”) are matters of ongoing controversy — especially returning genetic results for adult-onset conditions that are not clinically actionable in childhood [8, 9]. The debate intensified with the 2013 publication of recommendations from the American College of Medical Genetics and Genomics (ACMG) advising that clinicians notify their patients, regardless of age, when a variant known or expected to increase disease risk was identified incidentally through clinical sequencing in one of 56 clinically actionable genes not related to the test indication [10, 11]. Examples of clinically actionable conditions included hereditary breast and ovarian cancer (HBOC) syndrome (*BRCA1/2*), Lynch syndrome (*MLH1*, *MSH2*, *MSH6* and *PMS2*), and familial hypercholesterolemia (*LDLR*, *APOB* and *PCSK9*), all of which have Centers for Disease Control and Prevention (CDC) tier-one level of evidence for reducing morbidity and mortality in certain indications [12]. Of the 59 genes currently considered by the ACMG to be sufficiently actionable to merit patient analysis and notification, 52 are associated with conditions that have pediatric-onset or initiation of recommended risk reducing procedures in childhood [10, 13]. The remaining seven genes and their three associated conditions — HBOC (*BRCA1/2)*, Lynch syndrome (*MLH1, MSH2, MSH6, PMS2)*, and *MUTYH*-associated polyposis (*MUTYH*) — do not typically lead to pediatric onset of disease [10, 13], and thus, recommended surveillance and risk-reducing actions are postponed until adulthood [14, 15].

### Opposition to disclosure of adult-onset, clinically actionable results to minors

ACMG’s recommendations and subsequent reaffirmations regarding disclosure of secondary findings regardless of age [10, 11, 13] contrast with long-standing recommendations and policy statements by professional societies — including the ACMG [16] — to defer clinical testing for adult-onset genetic conditions until minors reach adulthood and can decide for themselves whether to have testing. Professional guidelines recommending against testing for adult-onset genetic conditions are based on expert consensus and are focused on the traditional normative standard — the best interests of the minor — and cite concern about potential harms as well as absence of clear medical benefit in childhood [16–23]. Potential harms and wrongs include psychological impacts such as increased distress, negative impacts on self-image, feelings of guilt or blame towards a family member, and misattributing symptoms to the condition [16, 24–26]. Additionally, disclosing an adult-onset genetic result to a minor and their parent could disrupt family relationships through differential treatment by parents (including “vulnerable child syndrome”) or increased parental anxiety and/or guilt [18, 24–26]. Of further concern are the potential for discrimination by life or disability insurers and stigmatization by peers [24, 25]. Finally, some scholars have suggested that childhood testing fails to respect the minor’s future autonomy by infringing upon their “right to an open future” in which they can decide for themselves whether or not to be tested [17, 18, 24–26]. These ethical arguments underpinning the professional guidelines regarding genetic testing in childhood are reviewed extensively elsewhere [18, 22, 24, 25].

### Support for disclosure of adult-onset, clinically actionable results to minors

In contrast, authors of the ACMG secondary finding recommendations and other proponents of returning actionable clinical or research findings to all patients, regardless of age, advocate for the broader interests of the family and of the minor to be included in the risk-benefit analysis [27]. For instance, they say, identifying an adult-onset condition in a minor could prompt adult relatives, including parents, to be tested for a potentially life-threatening condition (hereafter referred to as “cascade testing”), thereby protecting the interests of dependent minors [10, 28]. Other proposed benefits of disclosing adult-onset genetic results to minors include psychological benefits (e.g., the opportunity to adjust to hereditary disease), the ability to inform life planning (e.g., reproductive decision-making), and positive impact on family relationships (e.g., promotion of realistic parental expectations) [24, 26]. Additionally, some argue that disclosing adult-onset, clinically actionable results promotes autonomy, given that parents are best placed to decide what is in their child’s best interest [24], adolescents can contribute to informed decision-making [24], and failing to disclose the variant could prevent families from ever knowing their risk and, therefore, could deny the minor the opportunity to know about their risk in adulthood [27, 29]. Finally, there could be legal incentives to disclose clinically actionable variants to minors in states where courts recognize the “loss of chance” doctrine [30, 31], a medical malpractice doctrine that enables a plaintiff (patient) to bring suit against a defendant (medical provider) whose breach of duty substantially reduced the chance of a more favorable outcome (such as a delayed diagnosis diminishing the chance of recovery from a pre-existing medical condition such as a variant conferring genetic risk). This protocol paper focuses on the research components involving human participants. The PROGRESS study team also will be conducting legal research regarding the loss of chance doctrine that will be discussed separately.

### Parent and adolescent stakeholder views

While genetics providers, laboratories, and ethicists have debated disclosure of clinically actionable results to minors and their parents, empirical studies have found interest by parents and adolescents in receiving genetic findings even if the minor’s health care is not immediately affected [32–39]. For example, half of a sample of British adults felt that parents should be able to test their children for adult-onset conditions, even while acknowledging the validity of reasons for deferring testing until adulthood (e.g., stigma, fear of discrimination) [38]. Nearly all participants in focus groups of parents of pediatric participants in Geisinger’s MyCode® Community Health Initiative wanted Lynch syndrome results for their children, explaining that the importance of these results to their children’s future health outweighed the right of minors to make their own testing decisions once they reach adulthood [35]. Adolescents in several studies of stakeholders’ views of receiving results from genome-scale sequencing also expressed interest in adult-onset results and in being involved in decision-making about whether to learn these results [33, 36, 37, 40]. Furthermore, student participants in the 2016 American Society of Human Genetics (ASHG) DNA Day Essay Contest were asked to name an adult-onset genetic condition and defend or refute ASHG’s 2015 recommendation [17] to defer testing for adult-onset conditions until adulthood. Of the 205 students who wrote about HBOC syndrome, 56% argued for *BRCA1/2* testing before adulthood, citing reasons such as prevention and life planning [39].

As Mand et al. [24] note, “[m] ost arguments on both sides are testable empirical claims, so far untested, rather than abstract ethical or philosophical positions.” The limited evidence that does exist from minors who underwent genetic testing has not substantiated the negative psychosocial impacts anticipated by those opposed to the return of genetic information prior to adulthood [41, 42]. Specific to a clinically actionable, adult-onset condition, one study found that, female adolescents (age 11–19 years) from *BRCA1/2* families did not differ in their general psychosocial adjustment as compared to girls from breast cancer families without a *BRCA1/2* pathogenic/likely pathogenic variant and peers without breast cancer in their family [43]. However, the available evidence concerning minors’ psychosocial outcomes after receiving their own genetic results is limited by a general focus on pediatric- rather than adult-onset conditions, methodological differences that hinder comparisons, and a lack of longitudinal follow-up that would facilitate a clear understanding of how adult-onset genetic findings affect minors and their families over time [41, 42, 44]. There is less evidence still about the optimal way of disclosing adult-onset genetic risks to minors and their parents, should evidence about the risks and benefits of disclosure suggest such a policy.

## Methods/design

The Pediatric Reporting of Genomic Results Study (PROGRESS) seeks to determine how best to use genetic information to guide care over the course of a minor’s development in ways that maximize the physical and psychosocial health of the minor and their family. Specifically, the study aims to use a mixed-methods, longitudinal, observational cohort study to:

***Aim 1:****Determine whether anxiety, depression, family functioning, and health-related quality of life differ at 12 months post-disclosure* among adolescents *(participants age 11-17), as well as among parents of minors (participants age 0-17) who: 1) receive an adult-onset result; 2) receive a pediatric-onset result; or 3) do not receive a genetic result.*

**Aim 2:***Assess cascade testing uptake and initiation of risk reduction behaviors among parents from whom the minor inherited their adult- or pediatric-onset genetic variant.*

Based on the limited available literature on the effects of informing minors about their genetic condition or their hereditary risk, we hypothesize that there will be no differences in primary psychosocial outcomes in adolescents and parents of minors who receive an adult-onset finding, those who receive a pediatric-onset finding, and those who do not receive a genetic finding.

### Geisinger’s MyCode® community health initiative

PROGRESS will leverage experience from reporting clinically actionable genetic findings to adults enrolled in Geisinger’s MyCode® Community Health Initiative (MyCode). As described elsewhere [45–47], Geisinger’s MyCode project was launched in 2007 and serves as a repository of blood, DNA, and serum samples from participants who consent to broad, health-related research use of their samples, including genomic analysis [48]. MyCode is a major resource for research that combines information obtained from DNA and serum with health information from the electronic health record and other sources with the intention of improving the prevention, diagnosis, and treatment of disease [47]. In 2012, MyCode began enrolling minors with parental or legal guardian consent and assent for enrollees age 7–17 years [47]. In 2013, Geisinger began developing a process to return clinically actionable results to adult MyCode participants through the Genomic Screening and Counseling Program (GSCP) [46, 49]. This study will augment the existing GSCP to return clinically actionable results to minors and their parents, while collecting data to assess psychological and behavioral outcomes among the participants and their parents who receive a genetic result.

### Group definitions

**Fig. 1:**
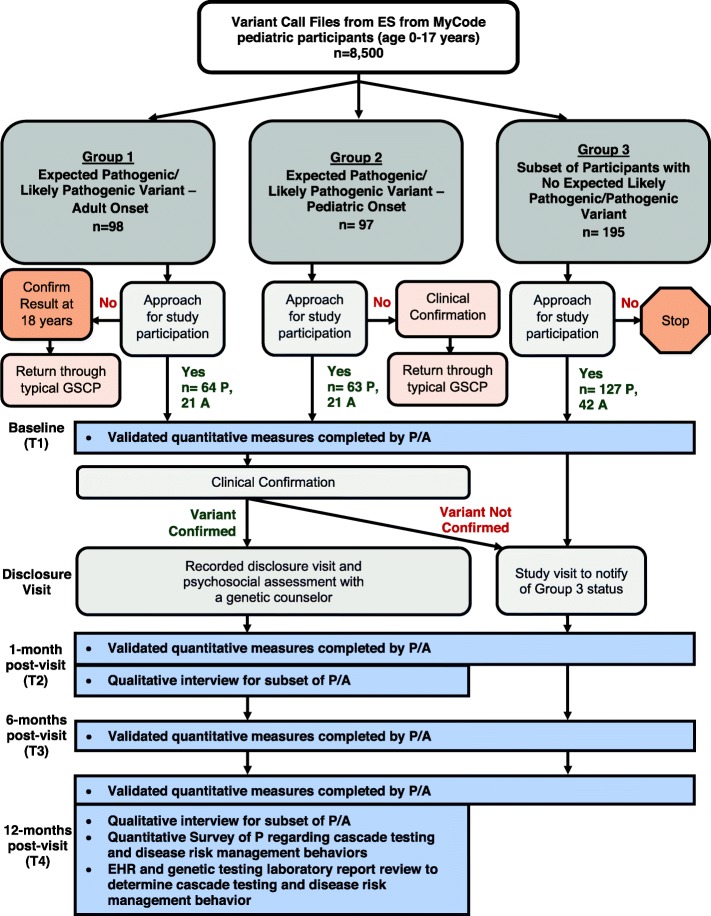
PROGRESS flow diagram. P=Parent of minor (ages 0–17), A = Adolescent (ages 11–17), ES = exome sequencing

Figure 1 summarizes the PROGRESS schema, which was approved by the Geisinger Institutional Review Board (IRB# 2018–0419). PROGRESS will use a mixed-methods, longitudinal, observational cohort study design to compare psychological outcomes and health-related quality of life among three groups of pediatric MyCode participants and their parent(s):

**Group 1** - Those with a clinically confirmed, clinically actionable, pathogenic/likely pathogenic variant in a gene associated with one of four adult-onset diseases for which no risk-reducing interventions are available in childhood — HBOC, Lynch syndrome, *MUTYH*-associated polyposis, and *HFE*-Associated Hereditary Hemochromatosis.

**Group 2** - Those with a clinically confirmed, clinically actionable, pathogenic/likely pathogenic variant in a gene associated with pediatric-onset disease or with adult-onset disease for which risk reducing interventions begin in childhood — all other ACMG SF v2.0 genes (Additional File 1).

**Group 3** - Those who do not have a potential pathogenic/likely pathogenic variant identified, and therefore do not receive a genetic result. Members of this group, who will be frequency matched to Group 1 and 2 participants based on age (+/- 2 years) and sex assigned at birth, will serve as controls to assess outcomes among members of Groups 1 and 2.

### Recruitment/enrollment

Variant files from exome sequencing completed through the DiscovEHR collaboration with Regeneron Genetics Center [48] for any pediatric MyCode participants between the age of 0–17 years will be assessed for pathogenic/likely pathogenic variants in 60 genes designated as actionable by MyCode (Additional File 1) [50]. This gene list includes the ACMG SF v2.0 list as well as biallelic *HFE* C282Y variants [13] (Additional File 1). Before clinical confirmation of variants in a CLIA-certified laboratory, a list of prospective pediatric participants will be generated. Prospective participants will include minors with a potential pathogenic/likely pathogenic variant (Groups 1 and 2) and age- (+/− 2 years) and biological sex-matched controls without such a variant (Group 3).

The study team will mail the parents of these prospective participants a letter describing the study, elements of informed consent, and an opportunity to opt out of additional study contact. Two weeks later, research staff will call those who have not opted out of study contact and offer an in-person visit to discuss the study. These staff, who will be blinded to potential participants’ expected study group, will lead the in-person consent process and obtain written documentation of parental consent. Prospective participants and their parents will be unaware of their potential group status during recruitment and enrollment. Pediatric participants ages 7–17 years will be engaged in the discussion and have the opportunity to provide assent. If an additional sample is required for MyCode study participation or clinical confirmation of a potential pathogenic/likely pathogenic variant [47, 49], this will be collected at the time of the study consent/assent visit. At the time of enrollment, study staff will also ask parents for guidance on how to disclose any results to their assenting children (e.g., at the in-person disclosure consultation or at a separate consult). If a minor is unable to assent due to such individual factors as a cognitive impairment, their parent(s) will be asked to consent, and if consent is obtained, the parent(s) will be included in the study. Participating minors who reach the age of majority (18 years) during the study will have the opportunity to participate in an informed consent process at age 18. Participants will be compensated for study participation after each completed quantitative survey. A subset of parents and adolescents will be invited to complete semi-structured interviews and will be compensated further.

### Exclusion criteria

Parents who decline participation and/or minors who do not assent and their parents will be excluded from the study. If assent/consent for PROGRESS are not given and the minor is suspected to have an adult-onset result, their sample will be held until the individual reaches 18 years of age and has re-consented to MyCode. If a minor is suspected to have a pediatric-onset result but consent/assent for PROGRESS are not given, their sample will proceed to clinical confirmation and, if confirmed, will follow established return procedures of the MyCode GSCP without further quantitative or qualitative data collection. Minors with an already identified genetic result for one of the 60 genes designated as actionable will be excluded when generating the list of potential participants. Minors who have not undergone genetic testing but have a known family history of a clinically actionable variant in one of the 60 genes will be eligible to participate. Minors who have already undergone exome sequencing on a clinical basis will be excluded from Group 1 or 2 if a variant in one of the 60 genes was identified and will also be excluded from Group 3 in light of their experience with genetic testing and potentially complex medical history.

### Sample size

Based on the current and anticipated pediatric participation in MyCode over the course of study enrollment, we estimate that 8500 minors will be eligible for the study. Given the expected rate of individuals with a pathogenic/likely pathogenic variant in one of the target genes — 2.3% of adult MyCode participants sequenced to date [50] — we anticipate 195 pediatric participants will have a genetic result. Ninety-eight (98) and 97 of these are anticipated to be in Groups 1 (adult-onset result) and 2 (pediatric-onset result), respectively. Based on experience recruiting MyCode participants for additional studies, we estimate that 65% of the families approached will consent to participate (internal data), leaving an estimated 64 minors in Group 1 and 63 in Group 2. Assuming conservatively that one parent enrolls for each child, we anticipate there will be 64 parents in Group 1 and 63 parents in Group 2. Since roughly one-third of the minors receiving a result will be age 11–17 years, and therefore eligible to contribute to data collection, we anticipate an additional 42 adolescents in Groups 1 and 2 (21 in each group). The eligible pool of pediatric MyCode participants with no genetic results to return will be matched on an age (+/− < 2 years) and biological sex distribution to Groups 1 and 2. We will approach 195 parents for inclusion in Group 3 and anticipate 65% to consent for participation (*n* = 127). We also anticipate an additional 42 adolescent participants in this group, for a total sample size of 254 parents and 84 adolescent participants across all three study groups.

For psychosocial outcomes in Aim 1, we have specified a priori each pairwise comparison to be of interest. Therefore, all calculations assume 80% power and 5% significance level. Using the sample sizes noted above with a 10% dropout, the minimum detectable effect size (change in standard deviation units) for the key quantitative psychosocial outcomes is 0.53, 0.45, and 0.46 for the comparison of Group 1 vs. Group 2, Group 1 vs. Group 3, and Group 2 vs. Group 3, respectively. If we are successful in recruiting a second parent for some of the minors, then we can expect the minimum detectable difference to decrease. These effect sizes are considered moderate in size and are less than the effect size seen in a previous study that used the Hospital Anxiety and Depression scale in a sample of adolescent girls from families with *BRCA1/2* variants [43].

The primary outcomes in Aim 2 are cascade testing uptake and initiation of recommended risk reduction. From the literature on cascade testing uptake in male and female first-degree relatives of individuals with a genetic condition [51–53], we estimate that approximately 50% of parents will complete cascade testing. To account for the possibility that cascade testing in one parent will spur uptake or negate the need for testing in another, we will incorporate an intra-class correlation value of 0.20. Based on the above sample size estimates, the study will be able to detect a 21% difference in the percentage of parents in Groups 1 compared to those in Group 2 that complete cascade testing (e.g., 50% vs. 73%). Based on a previous study among unaffected women with a pathogenic *BRCA1/2* variant [54] and assuming that males pursue management behaviors at a similar rate, we estimate that 65% of the parents will initiate risk reduction. Therefore, the study should be able to detect an 18% difference in the percentage of parents in Groups 1 compared to those in Group 2 who initiate risk reduction (e.g., 65% vs. 85%).

### Clinical confirmation and results disclosure

After consent/assent, DNA samples from participants with a potential pathogenic/likely pathogenic variant will be sent to a CLIA-certified clinical laboratory for confirmation [55]. Parents of minors with a clinically confirmed pathogenic/likely pathogenic result in one of the actionable genes of interest will learn of their child’s result during an in-person consultation conducted by a genetic counselor. Whether the minor learns of the result at the same disclosure consult as their parent(s) or during a separate consult will be dictated by the selections that the parent and minor made at the time of enrollment. Parents of minors whose variants are not confirmed clinically and participants without a pathogenic/likely pathogenic variant (Group 3) will be scheduled for a study visit to notify them of their study group assignment and remind them to follow-up with their healthcare providers if they have significant personal or family history of cancer or cardiovascular disease.

### Data collection

Data will be gathered via quantitative surveys using validated measures, qualitative interviews with adolescents and parents of minors, and review of electronic health record and testing laboratory data to determine parents’ cascade testing uptake and initiation of risk reducing behaviors (Table 1).

**Table 1:** Outcomes, covariates, time points and validated measures in quantitative surveys

**Data Collection Method**	**Domain**	**Validated Measure**	**Time Points**	**Completed By**
	**Aim 1 – Psychosocial Outcomes among Study Groups**			
	**Outcomes – All Groups**			
Quantitative survey	General anxiety and depression^a^	Assessment of symptoms of DSM-IV anxiety and depression in children [56] Hospital Anxiety & Depression Scale [57]	T1, T2, T3, T4	P/A
	Family functioning and cohesion^a^	General Functioning subscale (short form) of McMaster Family Assessment Device [58]	T1, T2, T3, T4	P/A
	Health-related quality of life^a^	CDC HRQOL– 4 [59]	T1, T2, T3, T4	P/A
	Body image	Body Image Scale [60]	T1, T2, T3, T4	A
	Self-esteem	Rosenberg Self-Esteem Scale [61]	T1, T2, T3, T4	A
	Decision regret	Decision Regret Scale [62]	T2, T4	P
	Patient satisfaction	Genetic Counseling Satisfaction [63]	T2	P
	**Covariates – All Groups**			
	Psychological flexibility	Acceptance and Action Questionnaire – II [64] Avoidance and Fusion Questionnaire for Youth [65]	T1, T2, T3, T4	P/A
	Lifestyle behaviors	Physical activity, diet, smoking and vaping [66] Alcohol consumption [67]	T1, T3, T4	A
	Information seeking	Health Information Orientation Scale [68]	T1	P
	Personal utility	Perceived utility of whole genome sequencing [69]	T1, T2, T3, T4	P
	Perceived risk	Perceived cancer/heart disease risk [43, 70]	T1, T2, T3, T4	P/A
	Health literacy	Brief health literacy scale [71]	T1	P
	Genomic literacy	Knowledge of genome sequencing [72]	T1	P
	**Outcomes – Participants with Genomic Result – Groups 1 and 2**			
	Condition specific distress	Children’s Revised Impact of Events Scale [73]	T2, T3, T4	P/A
	Adjustment to genetic information	Psychological adaptation to genetic information scale [74]	T2, T3, T4	P
	Patient education and empowerment	Health Education Impact Questionnaire [75]	T2, T3, T4	P
	Family communication	Family communication of genetic test results [76]	T3	P
Qualitative interview	Constructs for which validated measures do not exist (e.g. vulnerable child syndrome, right to an open future)	n/a	T2, T4	P/A
Psychosocial assessment			Disclosure Visit	P/A
Observation of reactions to disclosure			Disclosure Visit	P/A
	**Aim 2 – Cascade Testing and Risk reduction Initiation among Group 1 and 2 Parents**			
Quantitative Survey	Cascade testing uptake^a^	Adapted from family communication of genetic test results [76]	T4	P
	Initiation of risk management behaviors^a^	Adapted from risk management in unaffected women with pathogenic *BRCA1/2* variants [54]		
EHR review	Cascade testing uptake^a^	n/a	T4	n/a
	Initiation of risk management behaviors^a^	n/a		

^a^Primary outcomes, T1 = baseline; T2 = 1-month post-disclosure; T3 = 6-months post-disclosure; T4 = 12-months post-disclosure, P=Parent of minor (ages 0–17), A = Adolescent (ages 11–17), EHR = Electronic Health Record

Parent-participants will be asked to assess psychosocial outcomes for themselves and for their children. Adolescents will also participate in quantitative surveys and qualitative interviews. Adolescents who are unable to assent due to individual factors will be excluded from quantitative and qualitative measures.

### Quantitative measures

Survey instruments that include published quantitative measures (including those for anxiety/depression, psychological flexibility, family functioning, quality of life, body image, self-esteem, decisional regret, perceived cancer/heart disease risk, genetic counseling satisfaction, health literacy and genomic literacy) will be administered at the time of enrollment (T1, Additional Files 2 and 3), one-month post disclosure/visit (T2, Additional Files 4 and 5), six-months post disclosure/visit (T3), and/or 12 months post disclosure/visit (T4) for all three study groups [43, 54, 56–72, 76]. Additionally, Groups 1 and 2 will complete measures of condition-specific distress, adjustment to genetic information, family communication of genetic test results, and patient education and empowerment one-month post disclosure (T2), six-months post disclosure (T3), and/or 12-months post disclosure (T4) [73–75]. Longitudinal evaluation of a subset of these measures will enable exploration of changes over time. Table 1 summarizes the primary outcomes, covariates, and published measures collected in each study group. To ensure a satisfactory response rate, surveys will be offered via multiple modalities, including by phone, internet, and mail.

Additionally, parents of minors in Groups 1 and 2 will be surveyed at 12 months post-disclosure (T4) to determine whether parents of minors with a genetic result had cascade testing for the familial gene variant and whether those found to carry the familial variant have performed disease risk management behaviors (e.g., breast MRI for women with a pathogenic *BRCA1* variant). The study team will also query electronic health records to capture cascade testing and risk management behaviors among parents and will correspond with the genetic testing laboratory that confirmed the minor’s clinically actionable result to verify completion of cascade testing in the family.

### Qualitative measures

For Groups 1 and 2, the genetic counselor disclosing results will conduct a psychosocial assessment during the disclosure visit. Genetic counselors are qualified to conduct psychosocial assessments and provide brief psychosocial counseling [77]. The study clinical psychologists will review the genetic counselor’s approach to psychosocial assessments and provide input in accord with the psychologists’ expertise. The disclosure will be audio recorded for future qualitative review by the study team.

Semi-structured interviews with a subset of up to 45 participants (or until thematic saturation is achieved) will also be conducted by trained research staff using an interpretive phenomenological approach to elucidate the lived experience of adolescents and parents of minors receiving clinically actionable results [78]. Interviews will be conducted using an established interview guide with parents and adolescent participants from each group receiving results (Groups 1 and 2) at one-month (T2) and 12-months (T4) post disclosure (Additional Files 6 and  7). Approximately 15 interviews will be conducted among parents of younger children (age 0–10 years), 15 additional interviews will be conducted with parents of adolescents (age 11–17 years), and a final 15 interviews will be conducted among adolescents. The semi-structured format will enable data collection about pre-selected constructs for which established measures do not exist (such as “vulnerable child syndrome” and a “right to an open future”) while allowing participants to inform the study team of constructs that might not have been considered. Interviews will be conducted throughout the study’s duration to allow for assessment of changes in experience that could be related to modifications in practice for the target conditions (e.g., changes in risk management recommendations).

### Data analysis

Aim 1: Analyses will focus on understanding if change in the primary and secondary psychosocial outcomes from pre- to post-disclosure differs significantly among groups. The analysis of psychosocial change of the children will employ linear mixed models (LMMs) with random effects to capture correlation due to repeated measures. We will use the parental reporting for this analysis. The model will include random effects for the intercept and slope, and an interaction between the group indicator and time. If, after plotting the data, it is found that the slope of each outcome variable is not linear, then the random slope parameter will be replaced with a categorical, fixed-effects time variable. In either model parameterization, contrasts can be set up to test for change from baseline and compared among groups. A priori it is of interest to compare each group to the others; no post-hoc adjustment will be made. The groups will be compared on baseline covariates. If any are found to vary significantly, then the LMMs will be extended to include the potentially confounding variables. If any of the primary psychosocial outcomes are found to violate the normality assumption, we will consider transforming those variables or using Generalized Estimating Equations (GEEs). As a secondary analysis, we will analyze the responses of adolescents aged 11–17 using the same approaches as above. Additional analyses of the secondary outcomes will use regression models appropriate for a given distribution; LMM for continuous, logistic regression for binary/ordinal, and Poisson regression for discrete counts, all with including random effects to capture the within subject correlation due to repeated measures.

Aim 2: In this aim it is anticipated that any loss to follow-up will have minimal impact on the outcomes, as those data will be obtained from surveys at 12 months post-disclosure and via the electronic health record (EHR). Either self-report of cascade testing uptake or presence of cascade test result in the EHR will count as evidence of having had cascade testing.

Initiation of recommended disease risk reduction, a dichotomous variable, will be calculated for each parent -participant who is found to carry the familial gene variant. As with the assessment of cascade testing uptake, initiation of risk reduction will be determined by parental self-report at 12 months post-disclosure and via query of the Geisinger EHR and of Keystone Health Information Exchange. Participants will be considered to have performed recommended risk reduction, if at 12 months post-disclosure, they have had any of the risk reduction procedures recommended for individuals with their genetic condition. The analysis will use a random effects logistic regression model for cascade uptake. A random effect for family will be included to account for the inherent correlation of the clustered analysis design that collects data from parents. Comparisons between Groups 1 and 2 for initiation of recommended risk reduction will use a binary logistic regression model. Both models will include a covariate for Group membership. As described above under Aim 1, the models will be extended to include baseline covariates that were found to be different between groups.

### Psychosocial support

Given concerns about the potential for adverse psychosocial outcomes of returning adult-onset genetic results to minors [16–18, 22–24, 26], genetic counselors returning results, study staff administering instruments and scoring quantitative measures, and those performing qualitative interviews will notify the study’s pediatric clinical psychologists of any clinically relevant scale scores or psychological concerns that arise during data collection and/or results disclosure. Moreover, a clinical psychologist will check-in with all participants receiving a genetic result one-month post-disclosure (T2, Groups 1 and 2), will conduct periodic psychosocial assessments with adolescents with an adult-onset genetic result (Group 1), and will schedule separate therapeutic interactions with participants who exhibit clinically significant distress or other psychological outcomes. The study genetic counselor will also contact parents of children and adolescents at one- and six-months post-disclosure (T2, T3) to assess additional informational and support needs. Any unanticipated adverse events will be reported to the IRB and all adverse events (anticipated or unanticipated, serious or not, related or unrelated) will be reported to the funding agency.

Additionally, an external, five-member Event Monitoring Committee (EMC) [79, 80] has been convened to provide additional, independent study oversight and protection of the psychosocial wellbeing of pediatric participants. The EMC has multidisciplinary expertise relevant to the study (e.g., experts in adolescent health, bioethics, and pediatric genetics) and will work with the study team to address and prevent adverse events. In an effort to prevent adverse events, the EMC has reviewed study procedures and protocols and will have access to quantitative and qualitative data during the study to identify participant burden and psychosocial concerns. The EMC also will have the capacity to respond immediately to any serious adverse events, recommend changes to address or mitigate the impact of those events, and identify events that should lead to immediate cessation of the study. The EMC will provide additional, independent oversight to further safeguard pediatric participants’ welfare.

#### Study Status

As of March 16, 2020, 5212 pediatric participants have consented to MyCode and provided a sample for genomic analysis. Of those, 1878 have undergone exome sequencing as part of the DiscovEHR collaboration with Regeneron Genetics Center [48]. Review of research sequence data has shown that seven are eligible to be sent for clinical confirmation of an expected pathogenic/likely pathogenic variant in one of the 60 genes designated as actionable by MyCode. To date, seven parents of minors have been approached for the study; none have consented to participate.

## Discussion

Integrating exome and genome sequencing into clinical care and research has resulted in increasing opportunities to examine sequence data for pathogenic/likely pathogenic variants in clinically actionable genes. At present, there is a discrepancy between ACMG’s recommendation to return secondary findings without regard to age and various guidelines recommending against testing minors for adult-onset diseases due to concerns about negative impacts. Data are needed to inform this discussion and shape policies, protocols, and clinical care [16, 44, 81].

This mixed-methods, longitudinal, observational cohort study is designed to address this evidentiary gap. Psychosocial and behavioral data will allow for comparison of outcomes in adolescents and parents of minors who receive an adult-onset result, in those who receive a pediatric-onset result, and in those who do not receive a genetic result. This is the first study of which we are aware that will disclose adult-onset results to minors and their parents and compare outcomes among study groups with and without an adult-onset result in a real-world setting. This will provide several key opportunities to inform the debate regarding the disclosure of these results to minors and their parents through research and clinical testing (e.g., cascade testing, return of variants as secondary findings). First, the study will allow for examination of whether the psychological outcomes of adolescents and parents of minors receiving an adult-onset result through a supportive clinical encounter differ from outcomes among those who receive a pediatric-onset finding or those without a genetic finding. The study has also been designed to collect quantitative and qualitative data longitudinally, thereby allowing nuanced assessment of outcomes that have historically raised concerns among clinicians and ethicists (e.g., parents may treat their children as vulnerable, or actions taken in response to the result may restrict children’s life choices). The study also allows us to determine whether returning adult-onset results to minors does, in fact, promote cascade testing among parents and to describe behavioral outcomes among parents. Finally, data collected to address the study’s primary outcomes might also enable clinicians and researchers to proactively identify which parents and adolescents may benefit from additional supportive resources when receiving clinically actionable, adult-onset genetic results, should evidence about the risks and benefits of disclosure suggest such a policy.

Several limitations are inherent in the study design and population. The study population corresponds to the local population which, although socioeconomically diverse and geographically rural, is of primarily Northern European ancestry [47]. The age and sex distribution of minors receiving a result will reflect those in which a pathogenic/likely pathogenic variant is identified, and therefore might not mirror MyCode pediatric participants overall. Additionally, primary analyses will be conducted using sex assigned at birth; however, given that gender identity could affect psychosocial outcomes, gender identity will also be collected as part of the study. Although our study will contribute critical data, additional studies will need to replicate findings in other populations to resolve the debate of whether to provide adult-onset genetic findings to minors. Furthermore, the 12-month post disclosure follow-up for all participants might not provide sufficient time for some of the psychosocial outcomes to manifest. Similar studies with lengthier time frames would provide information about psychosocial impact as younger patients transition to decisional maturity and as older minors transition to adulthood.

In sum, the PROGRESS study will compare psychosocial outcomes over time among minors who receive an adult-onset genetic result and their parents, those who receive a pediatric-onset result, and those who do not receive a genetic result. It will also describe cascade testing and risk-reduction behaviors among parents of minors who receive a genetic result. The study will provide much-needed data on the risks and benefits of disclosing genetic results related to adult-onset conditions to minors and their parents, informing policy and practice in this contested area of genomic medicine.

## Supplementary information

**Additional file 1.** Conditions, Associated Genes, and Typical Onset. 

**Additional file 2.** T1 surveys for parents of minors (ages 0–17).

**Additional file 3.** T1 surveys for adolescents (ages 11–17).

**Additional file 4.** T2 surveys for parents of minors (ages 0–17).

**Additional file 5.** T2 surveys for adolescents (ages 11–17).

**Additional file 6.** Interview guide for semi-structured interviews with a subset of parents of minors (ages 0–17).

**Additional file 7.** Interview guide for semi-structured interviews with a subset of adolescents (ages 11–17).

## Data Availability

Not Applicable.

## Abbreviations

A: Adolescent (ages 11-17)
ACMG: American College of Medical Genetics and Genomics
ASHG: American Society of Human Genetics
CDC: Centers for Disease Control and Prevention
EHR: Electronic Health Record
EMC: Event Monitoring Committee
ES: Exome Sequencing
GEE: Generalized Estimating Equation
GSCP: Genomic Screening and Counseling Program
HBOC: Hereditary Breast and Ovarian Cancer
LMM: Linear Mixed Model
NHGRI: National Human Genome Research Institute
NIH: National Institutes of Health
P: Parent of minor (ages 0-17)
PROGRESS: Pediatric Reporting of Genomic Results Study
