# Supplementary material for: Pediatric reporting of genomic results study (PROGRESS): a mixed-methods, longitudinal, observational cohort study protocol to explore disclosure of actionable adult- and pediatric-onset genomic variants to minors and their parents
Source: BMC Pediatr. 2020 May 15;20:222. doi: 10.1186/s12887-020-02070-4 (PMC7227212; doi:10.1186/s12887-020-02070-4)
Supplement: Supplementary file 1 — Additional file 1. Conditions, Associated Genes, and Typical Onset. [file 12887_2020_2070_MOESM1_ESM.docx]

| **Supplementary Table 1. Conditions, Associated Genes, and Typical Onset** | | | | | | |
| --- | --- | --- | --- | --- | --- | --- |
| **Condition(s)** | **Gene** | | | | | **Onset** |
| Hereditary breast and ovarian cancer (HBOC) | *BRCA1* | | | *BRCA2* | | Adult |
| *MUTYH*-associated polyposis | *MUTYH** | | | | | Adult |
| Lynch Syndrome | *MLH1*  *MSH2* | | *MSH6*  *PMS2* | | | Adult |
| Hereditary Hemochromatosis | *HFE*** | | | | | Adult |
| Li-Fraumeni syndrome | *TP53* | | | | | Pediatric/adult |
| Peutz-Jeghers syndrome | *STK11* | | | | | Pediatric/adult |
| Familial adenomatous polyposis | *APC* | | | | | Pediatric/adult |
| Juvenile polyposis | *BMPR1A* | | | *SMAD4* | | Pediatric/adult |
| Von Hippel–Lindau syndrome | *VHL* | | | | | Pediatric/adult |
| Multiple endocrine neoplasia type 1 | *MEN1* | | | | | Pediatric/adult |
| Multiple endocrine neoplasia type 2 | *RET* | | | | | Pediatric/adult |
| PTEN hamartoma tumor syndrome | *PTEN* | | | | | Pediatric/adult |
| Hereditary paraganglioma-pheochromocytoma syndrome | *SDHD*  *SDHAF2* | | | | *SDHC*  *SDHB* | Pediatric/adult |
| Neurofibromatosis type 2 | *NF2* | | | | | Pediatric/adult |
| Ehlers-Danlos syndrome, vascular type | *COL3A1* | | | | | Pediatric/adult |
| Marfan syndrome  Loeys-Dietz syndrome  Familial thoracic aortic aneurysms and dissections | *FBN1*  *TGFBR1*  *TGFBR2* | | | | *SMAD3*  *ACTA2*  *MYH11* | Pediatric/adult |
| Hypertrophic cardiomyopathy  Dilated cardiomyopathy | *MYBPC3*  *MYH7*  *TNNT2*  *TNNI3*  *TPM1*  *MYL3* | | | *ACTC1*  *PRKAG2*  *GLA*  *MYL2*  *LMNA* | | Pediatric/adult |
| Catecholaminergic polymorphic ventricular tachycardia | *RYR2* | | | | | Pediatric/adult |
| Arrhythmogenic right ventricular cardiomyopathy | *PKP2*  *DSP*  *DSC2* | | *TMEM43*  *DSG2* | | | Pediatric/adult |
| Romano-Ward long-QT syndrome types 1, 2, and 3  Brugada syndrome | *KCNQ1*  *KCNH2* | | | | *SCN5A* | Pediatric/adult |
| Familial hypercholesterolemia | *LDLR*  *APOB* | | | | *PCSK9* | Pediatric/adult |
| Malignant hyperthermia susceptibility | *RYR1* | *CACNA1S* | | | | Pediatric/adult |
| Tuberous sclerosis complex | *TSC1*  *TSC2* | | | | | Pediatric |
| WT1-related Wilms tumor | *WT1* | | | | | Pediatric |
| Retinoblastoma | *RB1* | | | | | Pediatric |
| Ornithine Transcarbamylase Deficiency | *OTC* | | | | | Pediatric |
| Wilson disease | *ATP7B** | | | | | Pediatric |

* Biallelic variants only

**Biallelic c.845G>A, p.C282Y variants only
