# Supplementary material for: Pediatric reporting of genomic results study (PROGRESS): a mixed-methods, longitudinal, observational cohort study protocol to explore disclosure of actionable adult- and pediatric-onset genomic variants to minors and their parents
Source: BMC Pediatr. 2020 May 15;20:222. doi: 10.1186/s12887-020-02070-4 (PMC7227212; doi:10.1186/s12887-020-02070-4)
Supplement: Supplementary file 2 — Additional file 2. T1 surveys for parents of minors (ages 0–17). [file 12887_2020_2070_MOESM2_ESM.pdf]

Pediatric RoR Survey Adult Baseline

Thank you for agreeing to take part in this study. As a reminder, your participation is voluntary. We expect this survey to take about 30 minutes or less. Your responses will help us improve our program and understand the needs of our patients. You will be asked several questions. Some questions will require "yes" or "no" answers, some will be on a 1-5 scale, and other questions will have multiple answer choices. Everything you tell us will be kept confidential. This means that your responses will only be shared with research team members. When we write our report, nothing will identify you. Please be honest with your responses. You can say whatever you want- nothing will hurt our feelings and nothing you say will have a negative effect on your care. Please remember, you can decline to answer any question and you may end the survey at any time.

Please complete the survey below.

Thank you!

HRQOL-4: Healthy Days Measure

The first few questions are about your overall health.

1) 1. Would you say that in general your health is:

◯ Excellent ◯ Very Good ◯ Good ◯ Fair ◯ Poor ◯ Don't know

2) 2. Now thinking about your physical health, which includes physical illness and injury, for how many days during the past 30 days was your physical health not good?

◯ 0
◯ 1
◯ 2
◯ 3
◯ 4
◯ 5
◯ 6
◯ 7
◯ 8
◯ 9
◯ 10
◯ 11
◯ 12
◯ 13
◯ 14
◯ 15
◯ 16
◯ 17
◯ 18
◯ 19
◯ 20
◯ 21
◯ 22
◯ 23
◯ 24
◯ 25
◯ 26
◯ 27
◯ 28
◯ 29
◯ 30
◯ Don't know

3) 3. Now thinking about your mental health, which includes stress, depression, and problems with emotions, for how many days during the past 30 days was your mental health not good?

◯ 0
◯ 1
◯ 2
◯ 3
◯ 4
◯ 5
◯ 6
◯ 7
◯ 8
◯ 9
◯ 10
◯ 11
◯ 12
◯ 13
◯ 14
◯ 15
◯ 16
◯ 17
◯ 18
◯ 19
◯ 20
◯ 21
◯ 22
◯ 23
◯ 24
◯ 25
◯ 26
◯ 27
◯ 28
◯ 29
◯ 30
◯ Don't know

4) 4. During the past 30 days, for about how many days did poor physical or mental health keep you from doing your usual activities, such as self-care, work, or recreation?

◯ 0
◯ 1
◯ 2
◯ 3
◯ 4
◯ 5
◯ 6
◯ 7
◯ 8
◯ 9
◯ 10
◯ 11
◯ 12
◯ 13
◯ 14
◯ 15
◯ 16
◯ 17
◯ 18
◯ 19
◯ 20
◯ 21
◯ 22
◯ 23
◯ 24
◯ 25
◯ 26
◯ 27
◯ 28
◯ 29
◯ 30
◯ Don't know

Perceived Risk

5) In your opinion, compared to other [men/women] your age, what are your chances of developing [heart disease/cancer]?

◯ Much lower ◯ Lower ◯ Same ◯ Higher ◯ Much higher ◯ Don't know

Health Literacy

The next several questions are about your experience with healthcare and your thoughts on genetic testing in healthcare.

6) How often do you have problems learning about your medical condition because of difficulty understanding written information?

◯ Always ◯ Often ◯ Sometimes ◯ Occasionally ◯ Never

7) How confident are you filling out medical forms by yourself?

◯ Extremely ◯ Quite a bit ◯ Somewhat ◯ A little bit ◯ Not at all

8) How often do you have someone help you read hospital materials?

◯ Always ◯ Often ◯ Sometimes ◯ Occasionally ◯ Never

Information Seeking: Health Information Orientation Scale

Please mark how true each statement is for you.

	Not at all true	A little bit	Somewhat true	Quite a bit	Very much true
9) You like to gather as much information as you can before making a decision.	◯	◯	◯	◯	◯
10) You like to review information multiple times before making a decision.	◯	◯	◯	◯	◯
11) After you've made a decision, you continue to look for related information.	◯	◯	◯	◯	◯
12) You like to make decisions quickly.	◯	◯	◯	◯	◯
13) You have difficulty making sense of information from multiple sources.	◯	◯	◯	◯	◯
14) You fear that you might find out something you don't want to know.	◯	◯	◯	◯	◯
15) You feel overwhelmed by the amount of information available.	◯	◯	◯	◯	◯
16) You think it's the doctor's job to deal with information, not yours.	◯	◯	◯	◯	◯

Genomic Literacy

		True	False
17)	Healthy parents can have a child with an inherited disease.	◯	◯
18)	If your close relatives have diabetes or heart disease, you are more likely to develop these conditions.	◯	◯
19)	Some genetic disorders occur more often within particular ethnic groups.	◯	◯
20)	Most genetic disorders are caused by only a single gene.	◯	◯
21)	Once a genetic marker for a disorder is identified in a person, the disorder can usually be prevented or cured.	◯	◯
22)	A disease is only genetically determined if more than one family member is affected.	◯	◯
23)	Some genetic disorders occur later in adult life.	◯	◯
24)	A healthy lifestyle can prevent or lessen the negative consequences of having genetic predispositions to some diseases.	◯	◯
25)	The environment has little or no effect on how genes contribute to disease.	◯	◯

Personal Utility of Genomic Information

		Yes	Probably yes	No	Probably no
26)	Do you think your child's study results will accurately identify your disease risk?	○	○	○	○
27)	Do you think your child's study results will influence what treatment you receive for your current or future medical problems?	○	○	○	○
28)	Do you think your child's study results will influence decisions you make about your medical care?	○	○	○	○
29)	Do you think your child's study results will influence your or your child's reproductive decisions?	○	○	○	○
30)	Do you think your child's study results will influence what medications you take?	○	○	○	○
31)	Do you think your child's study results will influence your end-of-life planning?	○	○	○	○

Psychological Flexibility : AAQ-I

The next several questions ask about your thoughts and feelings overall.

		Never true	Very seldom true	Seldom true	Sometimes true	Frequently true	Almost always true	Always true
32)	Your painful experiences and memories make it difficult for you to live a life that you would value.	○	○	○	○	○	○	○
33)	You're afraid of your feelings.	○	○	○	○	○	○	○
34)	You worry about not being able to control your worries and feelings.	○	○	○	○	○	○	○
35)	Your painful memories prevent you from having a fulfilling life.	○	○	○	○	○	○	○
36)	Emotions cause problems in your life.	○	○	○	○	○	○	○

37)

It seems like most people are handling their lives better than you are. ○ ○ ○ ○ ○ ○ ○

38) Worries get in the way of your success. ○ ○ ○ ○ ○ ○ ○

Hospital General Anxiety & Depression Scale

These next few questions are about how you feel.

39) You feel tense or 'wound up':

○ Most of the time ○ A lot of the time ○ From time to time, occasionally ○ Not at all

40) You still enjoy the things you used to enjoy:

○ Definitely as much ○ Not quite so much ○ Only a little ○ Hardly at all

41) You get a sort of frightened feeling as if something awful is about to happen:

○ Very definitely and quite badly ○ Yes, but not too badly ○ A little, but it doesn't worry me
○ Not at all

42) You can laugh and see the funny side of things:

○ As much as I always could ○ Not quite so much now ○ Definitely not so much now
○ Not at all

43) Worrying thoughts go through your mind:

○ A great deal of the time ○ A lot of the time ○ From time to time but not too often ○ Only occasionally

44) You feel cheerful:

○ Not at all ○ Not often ○ Sometimes ○ Most of the time

45) You can sit at ease and feel relaxed:

○ Definitely ○ Usually ○ Not Often ○ Not at all

46) You feel as if you are slowed down:

○ Nearly all the time ○ Very often ○ Sometimes ○ Not at all

47) You get a sort of frightened feeling like 'butterflies' in the stomach:

○ Not at all ○ Occasionally ○ Quite often ○ Very often

Revised Children's Anxiety and Depression Scale

These questions are about your child's thoughts and feelings.

Please select the word that shows how often each of these things happens to your child. There are no right or wrong answers.

		Never	Sometimes	Often	Always
48)	Your child feels sad or empty.	◯	◯	◯	◯
49)	Your child worries when he/she thinks he/she has done poorly at something	◯	◯	◯	◯
50)	Your child feels afraid of being alone at home	◯	◯	◯	◯
51)	Nothing is much fun for my child anymore	◯	◯	◯	◯
52)	Your child worries that something awful will happen to someone in the family	◯	◯	◯	◯
53)	Your child is afraid of being in crowded places (like shopping centers, the movies, buses, busy playgrounds)	◯	◯	◯	◯
54)	Your child worries what other people think of him/her	◯	◯	◯	◯
55)	Your child has trouble sleeping	◯	◯	◯	◯
56)	Your child feels scared to sleep on his/her own	◯	◯	◯	◯
57)	Your child has problems with his/her appetite	◯	◯	◯	◯
58)	Your child suddenly becomes dizzy or faint when there is no reason for this	◯	◯	◯	◯
59)	Your child has to do some things over and over again (like washing hands, cleaning or putting things in a certain order)	◯	◯	◯	◯
60)	Your child has no energy for things	◯	◯	◯	◯
61)	Your child suddenly starts to tremble or shake when there is no reason for this	◯	◯	◯	◯
62)	Your child cannot think clearly	◯	◯	◯	◯
63)					

	Your child feels worthless	◯	◯	◯	◯
64)	Your child has to think of special thoughts (like numbers or words) to keep bad things from happening	◯	◯	◯	◯
65)	Your child thinks about death	◯	◯	◯	◯
66)	Your child feels like he/she doesn't want to move	◯	◯	◯	◯
67)	Your child worries that he/she will suddenly get a scared feeling when there is nothing to be afraid of	◯	◯	◯	◯
68)	Your child is tired a lot	◯	◯	◯	◯
69)	Your child feels afraid that he/she will make a fool of him/herself in front of people	◯	◯	◯	◯
70)	Your child has to do some things in just the right way to stop bad things from happening	◯	◯	◯	◯
71)	Your child feels restless	◯	◯	◯	◯
72)	Your child worries that something bad will happen to him/her	◯	◯	◯	◯

Family Functioning Assessment Scale

73) 1. Planning family activities is difficult because you misunderstand each other

◯ Strongly Agree ◯ Agree ◯ Disagree ◯ Strongly Disagree

74) In times of crisis you can turn to each other for support

◯ Strongly Agree ◯ Agree ◯ Disagree ◯ Strongly Disagree

75) You cannot talk to each other about the sadness you feel

◯ Strongly Agree ◯ Agree ◯ Disagree ◯ Strongly Disagree

76) Individuals are accepted for who they are

◯ Strongly Agree ◯ Agree ◯ Disagree ◯ Strongly Disagree

77) You avoid discussing your fears and concerns

◯ Strongly Agree ◯ Agree ◯ Disagree ◯ Strongly Disagree

78) You can express feeling for each other

◯ Strongly Agree ◯ Agree ◯ Disagree ◯ Strongly Disagree

79) There are lots of bad feelings in your family

◯ Strongly Agree ◯ Agree ◯ Disagree ◯ Strongly Disagree

80) You feel accepted for who you are

◯ Strongly Agree ◯ Agree ◯ Disagree ◯ Strongly Disagree

81) Making decisions is a problem for your family

◯ Strongly Agree ◯ Agree ◯ Disagree ◯ Strongly Disagree

82) You are able to make decisions about how to solve problems

◯ Strongly Agree ◯ Agree ◯ Disagree ◯ Strongly Disagree

83) You don't get along well

◯ Strongly Agree ◯ Agree ◯ Disagree ◯ Strongly Disagree

84) You confide in each other

◯ Strongly Agree ◯ Agree ◯ Disagree ◯ Strongly Disagree
