# Supplementary material for: Pediatric reporting of genomic results study (PROGRESS): a mixed-methods, longitudinal, observational cohort study protocol to explore disclosure of actionable adult- and pediatric-onset genomic variants to minors and their parents
Source: BMC Pediatr. 2020 May 15;20:222. doi: 10.1186/s12887-020-02070-4 (PMC7227212; doi:10.1186/s12887-020-02070-4)
Supplement: Supplementary file 3 — Additional file 3. T1 surveys for adolescents (ages 11–17). [file 12887_2020_2070_MOESM3_ESM.pdf]

Pediatric Ror Survey Adolescent Baseline

Thank you for agreeing to take part in this study. As a reminder, your participation is voluntary. We expect this survey to take about 30 minutes or less. Your responses will help us improve our program and understand the needs of our patients. You will be asked several questions. Some questions will require "yes" or "no" answers, some will be on a 1-5 scale, and other questions will have multiple answer choices. Everything you tell us will be kept confidential. This means that your responses will only be shared with research team members- we will not share any responses with your parents. When we write our report, nothing will identify you. Please be honest with your responses. You can say whatever you want- nothing will hurt our feelings and nothing you say will have a negative effect on your care. Please remember, you can decline to answer any question and you may end the survey at any time.

Please complete the survey below. Thank you!

HRQOL-4: Healthy Days Measure

The first few questions are about your overall health.

Would you say that in general your health is:

○ Excellent ○ Very Good ○ Good ○ Fair ○ Poor ○ Don't know

Now thinking about your physical health, which includes physical illness and injury, for how many days during the past 30 days was your physical health not good?

◯ Don't know
◯ 0
◯ 1
◯ 2
◯ 3
◯ 4
◯ 5
◯ 6
◯ 7
◯ 8
◯ 9
◯ 10
◯ 11
◯ 12
◯ 13
◯ 14
◯ 15
◯ 16
◯ 17
◯ 18
◯ 19
◯ 20
◯ 21
◯ 22
◯ 23
◯ 24
◯ 25
◯ 26
◯ 27
◯ 28
◯ 29
◯ 30

Now thinking about your mental health, which includes stress, depression, and problems with emotions, for how many days during the past 30 days was your mental health not good?

◯ Don't know
◯ 0
◯ 1
◯ 2
◯ 3
◯ 4
◯ 5
◯ 6
◯ 7
◯ 8
◯ 9
◯ 10
◯ 11
◯ 12
◯ 13
◯ 14
◯ 15
◯ 16
◯ 17
◯ 18
◯ 19
◯ 20
◯ 21
◯ 22
◯ 23
◯ 24
◯ 25
◯ 26
◯ 27
◯ 28
◯ 29
◯ 30

During the past 30 days, for about how many days did poor physical or mental health keep you from doing your usual activities, such as self-care, work, or recreation?

◯ Don't know
◯ 0
◯ 1
◯ 2
◯ 3
◯ 4
◯ 5
◯ 6
◯ 7
◯ 8
◯ 9
◯ 10
◯ 11
◯ 12
◯ 13
◯ 14
◯ 15
◯ 16
◯ 17
◯ 18
◯ 19
◯ 20
◯ 21
◯ 22
◯ 23
◯ 24
◯ 25
◯ 26
◯ 27
◯ 28
◯ 29
◯ 30

Perceived Risk

In your opinion, compared to other kids your age, what are your chances of developing [heart disease/cancer]?

◯ Much lower ◯ Lower ◯ Same ◯ Higher ◯ Much higher ◯ Don't know

Lifestyle Behaviors

The next few questions are about your health habits. Remember that we will not share any responses with your parents.

Diet

How many servings of fruit or vegetables do you eat each day?

◯ 1 serving ◯ 2 servings ◯ 3 or more servings ◯ None ◯ Don't know/not sure

Physical Activity

During the past 7 days, on how many days were you physically active for a total of at least 60 minutes per day? (Add up all the time you spent in any kind of physical activity that increased your heart rate and made you breathe hard some of the time.)

◯ 0 days ◯ 1 day ◯ 2 days ◯ 3 days ◯ 4 days ◯ 5 days ◯ 6 days ◯ 7 days

Smoking & Vaping

Do you currently smoke cigarettes?

◯ Yes ◯ No ◯ Don't know/ prefer not to answer

On average how many cigarettes do you smoke per day?

◯ Less than a half a pack (1-10) ◯ Less than a pack (10-20) ◯ A pack a day ◯ 1-2 packs a day
◯ more than 2 packs a day

The next question asks about electronic vapor products, such as JUUL, Vuse, MarkTen, and blu. Electronic vapor products include e-cigarettes, vapes, vape pens, e-cigars, e- hookahs, hookah pens, and mods.

Have you ever used an electronic vapor product?

◯ Yes ◯ No ◯ Don't know/ prefer not to answer

During the past 30 days, on how many days did you use an electronic vapor product?

◯ 0 days ◯ 1 or 2 days ◯ 3 to 5 days ◯ 6 to 9 days ◯ 10 to 19 days ◯ 20 to 29 days
◯ All 30 days

The next two questions ask about other tobacco products.

During the past 30 days, on how many days did you use chewing tobacco, snuff, dip, snus, or dissolvable tobacco products, such as Copenhagen, Grizzly, Skoal, or Camel Snus? (Do not count any electronic vapor products.)

◯ 0 days ◯ 1 or 2 days ◯ 3 to 5 days ◯ 6 to 9 days ◯ 10 to 19 days ◯ 20 to 29 days
◯ All 30 days

During the past 30 days, on how many days did you smoke cigars, cigarillos, or little cigars?

◯ 0 days ◯ 1 or 2 days ◯ 3 to 5 days ◯ 6 to 9 days ◯ 10 to 19 days ◯ 20 to 29 days
◯ All 30 days

Alcohol Intake

How often do you have a drink containing alcohol?

◯ Never ◯ Monthly or less ◯ 2-4 times a month ◯ 2-3 times a week ◯ 4 or more times a week
◯ Don't know/ prefer not to answer

How many standard drinks containing alcohol do you have on a typical day?

◯ 1 or 2 ◯ 3 or 4 ◯ 5 or 6 ◯ 7 or 9 ◯ 10 or more

How often do you have six or more drinks on one occasion?

◯ Never ◯ Less than monthly ◯ Monthly ◯ Weekly ◯ Daily or almost daily

Rosenberg Self-Esteem Scale (RSE)

The next few questions are about how you feel about yourself.

Please record the appropriate answer for each item, depending on whether you strongly agree, agree, disagree, or strongly disagree with it.

	Strongly agree	Agree	Disagree	Strongly disagree
Overall, you are satisfied with yourself.	◯	◯	◯	◯
At times you think you are no good at all.	◯	◯	◯	◯
You feel that you have a number of good qualities.	◯	◯	◯	◯
You are able to do things as well as most other people.	◯	◯	◯	◯
You feel that you do not have much to be proud of.	◯	◯	◯	◯
You feel useless at times.	◯	◯	◯	◯
You feel that you're a person of worth.	◯	◯	◯	◯
You wish you could have more respect for yourself.	◯	◯	◯	◯
Overall, you are inclined to think that you are a failure.	◯	◯	◯	◯
You take a positive attitude toward yourself.	◯	◯	◯	◯

Body Image Scale

Please answer the following questions about how you feel about your body.

	Not at all	A Little	Quite a Bit	Very Much
Have you been feeling self-conscious about your appearance?	◯	◯	◯	◯

Have you been dissatisfied with your appearance when dressed?	◯	◯	◯	◯
Do you find it difficult to look at yourself naked?	◯	◯	◯	◯
Do you avoid people because of the way you feel about your appearance?	◯	◯	◯	◯

Family Functioning Assessment Scale

The next questions are about your family.

Please select the degree to which you agree or disagree with these statements about your family.

Planning family activities is difficult because you misunderstand each other

◯ Strongly Agree ◯ Agree ◯ Disagree ◯ Strongly Disagree

In times of crisis you can turn to each other for support

◯ Strongly Agree ◯ Agree ◯ Disagree ◯ Strongly Disagree

You cannot talk to each other about the sadness you feel

◯ Strongly Agree ◯ Agree ◯ Disagree ◯ Strongly Disagree

Individuals are accepted for who they are

◯ Strongly Agree ◯ Agree ◯ Disagree ◯ Strongly Disagree

You avoid discussing your fears and concerns

◯ Strongly Agree ◯ Agree ◯ Disagree ◯ Strongly Disagree

You can express feeling for each other

◯ Strongly Agree ◯ Agree ◯ Disagree ◯ Strongly Disagree

There are lots of bad feelings in your family

◯ Strongly Agree ◯ Agree ◯ Disagree ◯ Strongly Disagree

You feel accepted for who you are

◯ Strongly Agree ◯ Agree ◯ Disagree ◯ Strongly Disagree

Making decisions is a problem for your family

◯ Strongly Agree ◯ Agree ◯ Disagree ◯ Strongly Disagree

You are able to make decisions about how to solve problems

◯ Strongly Agree ◯ Agree ◯ Disagree ◯ Strongly Disagree

You don't get along well

◯ Strongly Agree ◯ Agree ◯ Disagree ◯ Strongly Disagree

You confide in each other

◯ Strongly Agree ◯ Agree ◯ Disagree ◯ Strongly Disagree

Psychological Flexibility: Acceptance and Fusion Questionnaire for Youth (AFQ-Y) Functioning Assessment Scale

The next several questions ask about your thoughts and feelings overall.

We want to know more about what you think, how you feel, and what you do. Read each sentence. Then, select how true the statement is for you.

	Not at all True	A little True	Pretty True	True	Very True
Your life won't be good until you feel happy.	◯	◯	◯	◯	◯
Your thoughts and feelings mess up your life.	◯	◯	◯	◯	◯
If you feel sad or afraid, then something must be wrong with you.	◯	◯	◯	◯	◯
The bad things you think about yourself must be true.	◯	◯	◯	◯	◯
You don't try out new things if you're afraid of messing up.	◯	◯	◯	◯	◯
You must get rid of your worries and fears so you can have a good life.	◯	◯	◯	◯	◯
You do all you can to make sure you don't look dumb in front of other people.	◯	◯	◯	◯	◯
You try hard to erase hurtful memories from your mind.	◯	◯	◯	◯	◯
You can't stand to feel pain or hurt in your body.	◯	◯	◯	◯	◯
If your heart beats fast, there must be something wrong with me.	◯	◯	◯	◯	◯

You push away thoughts and feelings that you don't like.	○	○	○	○	○
You stop doing things that are important to you whenever you feel bad.	○	○	○	○	○
You do worse in school when you have thoughts that make you feel sad.	○	○	○	○	○
You say things to make you sound cool.	○	○	○	○	○
You wish you could wave a magic wand to make all your sadness go away.	○	○	○	○	○
You are afraid of your feelings.	○	○	○	○	○
You can't be a good friend when you feel upset.	○	○	○	○	○

Revised Children's Anxiety and Depression Scale

	Never	Sometimes	Often	Always
You feel sad or empty.	○	○	○	○
You worry when you think you have done poorly at something	○	○	○	○
You feel afraid of being on your own at home	○	○	○	○
Nothing is much fun anymore	○	○	○	○
You worry that something awful will happen to someone in your family	○	○	○	○
You are afraid of being in crowded places (like shopping centers, the movies, buses, busy playgrounds)	○	○	○	○
You worry what other people think of you	○	○	○	○
You have trouble sleeping	○	○	○	○
You feel scared to sleep on your own	○	○	○	○
You have problems with your appetite	○	○	○	○
You suddenly become dizzy or faint when there is no reason for this	○	○	○	○
You have to do some things over and over again (like washing hands, cleaning or putting things in a certain order)	○	○	○	○
You have no energy for things	○	○	○	○
You suddenly start to tremble or shake when there is no reason for this	○	○	○	○
You cannot think clearly	○	○	○	○
You feel worthless	○	○	○	○
You have to think of special thoughts (like numbers or words) to keep bad things from happening	○	○	○	○
You think about death	○	○	○	○
You feel like you don't want to move	○	○	○	○

You worry that you will suddenly get a scared feeling when there is nothing to be afraid of	◯	◯	◯	◯
You are tired a lot	◯	◯	◯	◯
You feel afraid that you will make a fool of yourself in front of people	◯	◯	◯	◯
You have to do some things in just the right way to stop bad things from happening	◯	◯	◯	◯
You feel restless	◯	◯	◯	◯
You worry that something bad will happen to you	◯	◯	◯	◯

What was your biological sex assigned at birth?

◯ Female ◯ Male ◯ Intersex ◯ None of these options describe me ◯ Unsure ◯ Prefer not to answer

Biological sex other

____

What term best expresses how you describe your gender identity?

◯ Man ◯ Woman ◯ Non-binary ◯ Transgender ◯ None of these describe me ◯ Unsure
◯ Prefer not to answer

Gender identity other

____

What term best represents how you think of your sexual orientation?

◯ Gay ◯ Lesbian ◯ Straight ◯ Bisexual ◯ None of these describe me ◯ Unsure
◯ Prefer not to answer

Sexual Orientation other

____
