# Supplementary material for: Pediatric reporting of genomic results study (PROGRESS): a mixed-methods, longitudinal, observational cohort study protocol to explore disclosure of actionable adult- and pediatric-onset genomic variants to minors and their parents
Source: BMC Pediatr. 2020 May 15;20:222. doi: 10.1186/s12887-020-02070-4 (PMC7227212; doi:10.1186/s12887-020-02070-4)
Supplement: Supplementary file 4 — Additional file 4. T2 surveys for parents of minors (ages 0–17). [file 12887_2020_2070_MOESM4_ESM.pdf]

Pediatric RoR Survey Adult 1 Month

Thank you for agreeing to take part in this study.

As a reminder, your participation is voluntary. We expect this survey to take about 30 minutes or less. Your responses will help us improve our program and understand the needs of our patients. You will be asked several questions. Some questions will require “Yes” or “No” answers, some will be on a 1-5 scale, and other questions will have multiple answer choices. Everything you tell us will be kept confidential. This means that your responses will only be shared with research team members. When we write our report, nothing will identify you. Please be honest with your responses. You can say whatever you want – nothing will hurt our feelings and nothing you say will have a negative effect on your care. Please remember, you can decline to answer any question and you may end the survey at any time.

Thank you for agreeing to take part in this study. As a reminder, your participation is voluntary. We expect this survey to take about 30 minutes or less. Your responses will help us improve our program and understand the needs of our patients. You will be asked several questions. Some questions will require “Yes” or “No” answers, some will be on a 1-5 scale, and other questions will have multiple answer choices. Everything you tell us will be kept confidential. This means that your responses will only be shared with research team members. When we write our report, nothing will identify you. Please be honest with your responses. You can say whatever you want – nothing will hurt our feelings and nothing you say will have a negative effect on your care. Please remember, you can decline to answer any question and you may end the survey at any time.

HRQOL-4: Healthy Days Measure

1) Would you say that in general your health is:

◯ Excellent ◯ Very Good ◯ Good ◯ Fair ◯ Poor ◯ Don't know

2) Now thinking about your physical health, which includes physical illness and injury, for how many days during the past 30 days was your physical health not good?

○ 0 ○ 1 ○ 2 ○ 3 ○ 4 ○ 5 ○ 6 ○ 7 ○ 8 ○ 9 ○ 10 ○ 11 ○ 12
○ 13 ○ 14 ○ 15 ○ 16 ○ 17 ○ 18 ○ 19 ○ 20 ○ 21 ○ 22 ○ 23 ○ 24
○ 25 ○ 26 ○ 27 ○ 28 ○ 29 ○ 30 ○ Don't know

3) Now thinking about your mental health, which includes stress, depression, and problems with emotions, for how many days during the past 30 days was your mental health not good?

○ 0 ○ 1 ○ 2 ○ 3 ○ 4 ○ 5 ○ 6 ○ 7 ○ 8 ○ 9 ○ 10 ○ 11 ○ 12
○ 13 ○ 14 ○ 15 ○ 16 ○ 17 ○ 18 ○ 19 ○ 20 ○ 21 ○ 22 ○ 23 ○ 24
○ 25 ○ 26 ○ 27 ○ 28 ○ 29 ○ 30 ○ Don't know

4) During the past 30 days, for about how many days did poor physical or mental health keep you from doing your usual activities, such as self-care, work, or recreation?

○ 0 ○ 1 ○ 2 ○ 3 ○ 4 ○ 5 ○ 6 ○ 7 ○ 8 ○ 9 ○ 10 ○ 11 ○ 12
○ 13 ○ 14 ○ 15 ○ 16 ○ 17 ○ 18 ○ 19 ○ 20 ○ 21 ○ 22 ○ 23 ○ 24
○ 25 ○ 26 ○ 27 ○ 28 ○ 29 ○ 30 ○ Don't know

Perceived Risk

5) In your opinion, compared to other [men/women] your age, what are your chances of developing [heart disease/cancer]?

○ Much Lower ○ Lower ○ Same ○ Higher ○ Much Higher ○ Don't Know

Do you think results from genetic testing will...

		Yes	Probably Yes	No	Probably No
6)	...accurately identify your disease risk?	○	○	○	○
7)	...influence what treatment you receive for your current or future medical problems?	○	○	○	○
8)	...influence decisions you make about your medical care?	○	○	○	○
9)	...influence your or your child's reproductive decisions?	○	○	○	○
10)	...influence what medications you take?	○	○	○	○
11)	...influence your end-of-life planning?	○	○	○	○

Please rate how true each statement is for you by selecting one of the following numbers: 1 - never true, 2 - very seldom true, 3 - seldom true, 4 - sometimes true, 5 - frequently true, 6 - almost always true, 7 - always true.

	1 Never True	2 Very Seldom True	3 Seldom True	4 Sometimes True	5 Frequently True	6 Almost Always True	7 Always True
12) Your painful experiences and memories make it difficult for you to live a life that you would value.	◯	◯	◯	◯	◯	◯	◯
13) You're afraid of your feelings.	◯	◯	◯	◯	◯	◯	◯
14) You worry about not being able to control your worries and feelings.	◯	◯	◯	◯	◯	◯	◯
15) Your painful memories prevent you from having a fulfilling life.	◯	◯	◯	◯	◯	◯	◯
16) Emotions cause problems in your life.	◯	◯	◯	◯	◯	◯	◯
17) It seems like most people are handling their lives better than you are.	◯	◯	◯	◯	◯	◯	◯
18) Worries get in the way of your success.	◯	◯	◯	◯	◯	◯	◯

Hospital General Anxiety & Depression Scale

19) You feel tense or 'wound up':

◯ Most of the time ◯ A lot of the time ◯ From time to time, occasionally ◯ Not at all

20) You still enjoy the things you used to enjoy:

◯ Definitely as much ◯ Not quite so much ◯ Only a little ◯ Hardly at all

21) You get a sort of frightened feeling as if something awful is about to happen:

◯ Very definitely and quite badly ◯ Yes, but not too badly ◯ A little, but it doesn't worry me
◯ Not at all

22) You can laugh and see the funny side of things:

◯ As much as you always could ◯ Not quite so much now ◯ Definitely not so much now
◯ Not at all

23) Worrying thoughts go through your mind:

◯ A great deal of the time ◯ A lot of the time ◯ From time to time but not too often ◯ Only occasionally

24) You feel cheerful:

◯ Not at all ◯ Not often ◯ Sometimes ◯ Most of the time

25) You can sit at ease and feel relaxed:

◯ Definitely ◯ Usually ◯ Not Often ◯ Not at all

26) You feel as if you slowed down:

◯ Nearly all the time ◯ Very often ◯ Sometimes ◯ Not at all

27) You get a sort of frightened feeling like 'butterflies' in the stomach:

◯ Not at all ◯ Occasionally ◯ Quite often ◯ Very often

These questions are about your child's thoughts and feelings.

		Never	Sometimes	Often	Always
28)	Your child feels sad or empty.	○	○	○	○
29)	Your child worries when he/she thinks he/she has done poorly at something	○	○	○	○
30)	Your child feels afraid of being alone at home	○	○	○	○
31)	Nothing is much fun for your child anymore	○	○	○	○
32)	Your child worries that something awful will happen to someone in the family	○	○	○	○
33)	Your child is afraid of being in crowded places (like shopping centers, the movies, buses, busy playgrounds)	○	○	○	○
34)	Your child worries what other people think of him/her	○	○	○	○
35)	Your child has trouble sleeping	○	○	○	○
36)	Your child feels scared to sleep on his/her own	○	○	○	○
37)	Your child has problems with his/her appetite	○	○	○	○
38)	Your child suddenly becomes dizzy or faint when there is no reason for this	○	○	○	○
39)	Your child has to do some things over and over again (like washing hands, cleaning or putting things in a certain order)	○	○	○	○
40)	Your child has no energy for things	○	○	○	○
41)	Your child suddenly starts to tremble or shake when there is no reason for this	○	○	○	○
42)	Your child cannot think clearly	○	○	○	○
43)	Your child feels worthless	○	○	○	○
44)	Your child has to think of special thoughts (like numbers or words) to keep bad things from happening	○	○	○	○

45)

	Your child thinks about death	◯	◯	◯	◯
46)	Your child feels like he/she doesn't want to move	◯	◯	◯	◯
47)	Your child worries that he/she will suddenly get a scared feeling when there is nothing to be afraid of	◯	◯	◯	◯
48)	Your child is tired a lot	◯	◯	◯	◯
49)	Your child feels afraid that he/she will make a fool of him/herself in front of people	◯	◯	◯	◯
50)	Your child has to do some things in just the right way to stop bad things from happening	◯	◯	◯	◯
51)	Your child feels restless	◯	◯	◯	◯
52)	Your child worries that something bad will happen to him/her	◯	◯	◯	◯

53) Planning family activities is difficult because you misunderstand each other

◯ Strongly Agree ◯ Agree ◯ Disagree ◯ Strongly Disagree

54) In times of crisis you can turn to each other for support

◯ Strongly Agree ◯ Agree ◯ Disagree ◯ Strongly Disagree

55) You cannot talk to each other about the sadness you feel

◯ Strongly Agree ◯ Agree ◯ Disagree ◯ Strongly Disagree

56) Individuals are accepted for who they are

◯ Strongly Agree ◯ Agree ◯ Disagree ◯ Strongly Disagree

57) You avoid discussing your fears and concerns

◯ Strongly Agree ◯ Agree ◯ Disagree ◯ Strongly Disagree

58) You can express feeling for each other

◯ Strongly Agree ◯ Agree ◯ Disagree ◯ Strongly Disagree

59) There are lots of bad feelings in your family

◯ Strongly Agree ◯ Agree ◯ Disagree ◯ Strongly Disagree

60) You feel accepted for who you are

◯ Strongly Agree ◯ Agree ◯ Disagree ◯ Strongly Disagree

61) Making decisions is a problem for your family

◯ Strongly Agree ◯ Agree ◯ Disagree ◯ Strongly Disagree

62) You are able to make decisions about how to solve problems

◯ Strongly Agree ◯ Agree ◯ Disagree ◯ Strongly Disagree

63) You don't get along well

◯ Strongly Agree ◯ Agree ◯ Disagree ◯ Strongly Disagree

64) You confide in each other

◯ Strongly Agree ◯ Agree ◯ Disagree ◯ Strongly Disagree

Decision Regret

65) It was the right decision

◯ Strongly Agree ◯ Agree ◯ Neither Agree Nor Disagree ◯ Disagree ◯ Strongly Disagree

66) You regret the choice that was made

◯ Strongly Agree ◯ Agree ◯ Neither Agree Nor Disagree ◯ Disagree ◯ Strongly Disagree

67) You would go for the same choice again if you had to do it over again

◯ Strongly Agree ◯ Agree ◯ Neither Agree Nor Disagree ◯ Disagree ◯ Strongly Disagree

68) The choice did you a lot of harm?

◯ Strongly Agree ◯ Agree ◯ Neither Agree Nor Disagree ◯ Disagree ◯ Strongly Disagree

69) The decision was a wise one?

◯ Strongly Agree ◯ Agree ◯ Neither Agree Nor Disagree ◯ Disagree ◯ Strongly Disagree

Genetic Counseling Satisfaction

70) Your child's genetic counselor seemed to understand the stresses you were facing

◯ Strongly Disagree ◯ Disagree Somewhat ◯ Uncertain ◯ Agree Somewhat ◯ Agree Strongly

71) Your child's genetic counselor helped you to identify what you needed to know to make decisions about what would happen

◯ Strongly Agree ◯ Agree ◯ Neither Agree Nor Disagree ◯ Disagree ◯ Strongly Disagree

72) You felt better about your child's health after meeting with their genetic counselor

◯ Strongly Agree ◯ Agree ◯ Neither Agree Nor Disagree ◯ Disagree ◯ Strongly Disagree

73) The genetic counseling session was about the right length of time you needed

◯ Strongly Agree ◯ Agree ◯ Neither Agree Nor Disagree ◯ Disagree ◯ Strongly Disagree

74) The genetic counselor was truly concerned about your child's well-being

◯ Strongly Agree ◯ Agree ◯ Neither Agree Nor Disagree ◯ Disagree ◯ Strongly Disagree

75) The genetic counseling session was valuable to you

◯ Strongly Agree ◯ Agree ◯ Neither Agree Nor Disagree ◯ Disagree ◯ Strongly Disagree

Health Education Impact Questionnaire (heiQ)

76) You communicate very confidently with your doctor about your child's MyCode genetic test result

◯ Strongly Disagree ◯ Disagree ◯ Agree ◯ Strongly Agree

77) You have very positive relationships with your child's healthcare providers regarding their MyCode genetic test result

◯ Strongly Disagree ◯ Disagree ◯ Agree ◯ Strongly Agree

78) You confidently give healthcare professionals information about your child's MyCode genetic test result

◯ Strongly Disagree ◯ Disagree ◯ Agree ◯ Strongly Agree

79) You get your needs met from available healthcare resources for your child's MyCode genetic test result

◯ Strongly Disagree ◯ Disagree ◯ Agree ◯ Strongly Agree

80) You work in a team with your child's doctors and other healthcare providers for your child's MyCode genetic test result

◯ Strongly Disagree ◯ Disagree ◯ Agree ◯ Strongly Agree

Please check each item showing how frequently these comments were true for you during the past 7 days.

		Not at All	Rarely	Sometimes	Often
81)	Do you think about it even when you don't mean to?	○	○	○	○
82)	Do you try to remove it from your memory?	○	○	○	○
83)	Do you have difficulty paying attention or concentrating?	○	○	○	○
84)	Do you have waves of strong feelings about it?	○	○	○	○
85)	Do you startle more easily or feel more nervous than you did before it happened?	○	○	○	○
86)	Do you stay away from reminder of it (e.g. places or situations)?	○	○	○	○
87)	Do you try not to talk about it?	○	○	○	○
88)	Do pictures about it pop into your mind?	○	○	○	○
89)	Do other things keep making you think about it?	○	○	○	○
90)	Do you try not to think about it?	○	○	○	○
91)	Do you get easily irritable?	○	○	○	○
92)	Are you alert and watchful when there no obvious need to be?	○	○	○	○
93)	Do you have sleep problems?	○	○	○	○

Adjustment to Genetic Information

94) How upset did you feel about your child's MyCode genetic test result?

○ A Little ○ Somewhat ○ A Good Deal ○ A Great Deal

95) How anxious or nervous did you feel about your child's MyCode genetic test result?

○ A Little ○ Somewhat ○ A Good Deal ○ A Great Deal

96) How sad did you feel about your child's MyCode genetic test result?

○ A Little ○ Somewhat ○ A Good Deal ○ A Great Deal

97) How happy did you feel about your child's MyCode genetic test result?

○ A Little ○ Somewhat ○ A Good Deal ○ A Great Deal

98) How relieved did you feel about your child's MyCode genetic test result?

◯ A Little ◯ Somewhat ◯ A Good Deal ◯ A Great Deal

99) How much did you feel that you understood clearly your choices for disease prevention or early detection for your child?

◯ A Little ◯ Somewhat ◯ A Good Deal ◯ A Great Deal

100) How helpful was the information you received from your child's MyCode genetic test result in planning for the future?

◯ A Little ◯ Somewhat ◯ A Good Deal ◯ A Great Deal

101) How frustrated did you feel that there are no definite disease prevention guidelines for your child?

◯ A Little ◯ Somewhat ◯ A Good Deal ◯ A Great Deal

102) How uncertain did you feel about what your child's MyCode genetic test result means for them?

◯ A Little ◯ Somewhat ◯ A Good Deal ◯ A Great Deal

103) How uncertain did you feel about what your child's MyCode genetic test result means for your child's risk of disease?

◯ A Little ◯ Somewhat ◯ A Good Deal ◯ A Great Deal

104) How concerned did you feel that your child's MyCode genetic test result would affect your health insurance status?

◯ A Little ◯ Somewhat ◯ A Good Deal ◯ A Great Deal

105) How concerned did you feel that your child's MyCode genetic test result would affect your employment status?

◯ A Little ◯ Somewhat ◯ A Good Deal ◯ A Great Deal
