# Supplementary material for: Pediatric reporting of genomic results study (PROGRESS): a mixed-methods, longitudinal, observational cohort study protocol to explore disclosure of actionable adult- and pediatric-onset genomic variants to minors and their parents
Source: BMC Pediatr. 2020 May 15;20:222. doi: 10.1186/s12887-020-02070-4 (PMC7227212; doi:10.1186/s12887-020-02070-4)
Supplement: Supplementary file 5 — Additional file 5. T2 surveys for adolescents (ages 11–17). [file 12887_2020_2070_MOESM5_ESM.pdf]

Pediatric Ror Survey Adolescent 1 Month

Thank you for agreeing to take part in this study.

As a reminder, your participation is voluntary. We expect this survey to take about less than 30 minutes. Your responses will help us improve our program and understand the needs of our patients. You will be asked several questions. Some questions will require “Yes” or “No” answers, some will be on a 1-5 scale, and other questions will have multiple answer choices. Everything you tell us will be kept confidential. This means that your responses will only be shared with research team members – we will not share any responses with your parents. When we write our report, nothing will identify you. Please be honest with your responses. You can say whatever you want – nothing will hurt our feelings and nothing you say will have a negative effect on your care. Please remember, you can decline to answer any question and you may end the survey at any time.

HRQOL-4: Healthy Days Measure

1) Would you say that in general your health is:

○ Excellent ○ Very Good ○ Good ○ Fair ○ Poor ○ Don't know

2) Now thinking about your physical health, which includes physical illness and injury, for how many days during the past 30 days was your physical health not good?

○ 0 ○ 1 ○ 2 ○ 3 ○ 4 ○ 5 ○ 6 ○ 7 ○ 8 ○ 9 ○ 10 ○ 11 ○ 12
○ 13 ○ 14 ○ 15 ○ 16 ○ 17 ○ 18 ○ 19 ○ 20 ○ 21 ○ 22 ○ 23 ○ 24
○ 25 ○ 26 ○ 27 ○ 28 ○ 29 ○ 30 ○ Don't know

3) Now thinking about your mental health, which includes stress, depression, and problems with emotions, for how many days during the past 30 days was your mental health not good?

○ 0 ○ 1 ○ 2 ○ 3 ○ 4 ○ 5 ○ 6 ○ 7 ○ 8 ○ 9 ○ 10 ○ 11 ○ 12
○ 13 ○ 14 ○ 15 ○ 16 ○ 17 ○ 18 ○ 19 ○ 20 ○ 21 ○ 22 ○ 23 ○ 24
○ 25 ○ 26 ○ 27 ○ 28 ○ 29 ○ 30 ○ Don't know

4) During the past 30 days, for about how many days did poor physical or mental health keep you from doing your usual activities, such as self-care, work, or recreation?

○ 0 ○ 1 ○ 2 ○ 3 ○ 4 ○ 5 ○ 6 ○ 7 ○ 8 ○ 9 ○ 10 ○ 11 ○ 12
○ 13 ○ 14 ○ 15 ○ 16 ○ 17 ○ 18 ○ 19 ○ 20 ○ 21 ○ 22 ○ 23 ○ 24
○ 25 ○ 26 ○ 27 ○ 28 ○ 29 ○ 30 ○ Don't know

Perceived Risk

5) In your opinion, compared to other kids your age, what are your chances of developing [heart disease/cancer]?

○ Much lower ○ Lower ○ Same ○ Higher ○ Much higher ○ Don't know

Please tell us the appropriate answer for each item, depending on whether you strongly agree, agree, disagree, or strongly disagree with it.

		Strongly agree	Agree	Disagree	Strongly disagree
6)	Overall, you are satisfied with yourself.	○	○	○	○
7)	At times you think you are no good at all.	○	○	○	○
8)	You feel that you have a number of good qualities.	○	○	○	○
9)	You are able to do things as well as most other people.	○	○	○	○
10)	You feel you do not have much to be proud of.	○	○	○	○
11)	You feel useless at times.	○	○	○	○
12)	You feel that you're a person of worth.	○	○	○	○
13)	You wish you could have more respect for yourself.	○	○	○	○

14)

Overall, you are inclined to think that you are a failure.	◯	◯	◯	◯
15) You take a positive attitude toward yourself.	◯	◯	◯	◯

Please answer the following questions about how you feel about your body

	Not at all	A Little	Quite a Bit	Very Much
16) Have you been feeling self-conscious about your appearance?	◯	◯	◯	◯
17) Have you been dissatisfied with your appearance when dressed?	◯	◯	◯	◯
18) Do you find it difficult to look at yourself naked?	◯	◯	◯	◯
19) Do you avoid people because of the way you feel about your appearance?	◯	◯	◯	◯

Family Functioning Assessment Scale

20) Planning family activities is difficult because you misunderstand each other

◯ Strongly Agree ◯ Agree ◯ Disagree ◯ Strongly Disagree

21) In times of crisis you can turn to each other for support

◯ Strongly Agree ◯ Agree ◯ Disagree ◯ Strongly Disagree

22) You cannot talk to each other about the sadness you feel

◯ Strongly Agree ◯ Agree ◯ Disagree ◯ Strongly Disagree

23) Individuals are accepted for who they are

◯ Strongly Agree ◯ Agree ◯ Disagree ◯ Strongly Disagree

24) You avoid discussing your fears and concerns

◯ Strongly Agree ◯ Agree ◯ Disagree ◯ Strongly Disagree

25) You can express feeling for each other

◯ Strongly Agree ◯ Agree ◯ Disagree ◯ Strongly Disagree

26) There are lots of bad feelings in your family

◯ Strongly Agree ◯ Agree ◯ Disagree ◯ Strongly Disagree

27) You feel accepted for who you are

◯ Strongly Agree ◯ Agree ◯ Disagree ◯ Strongly Disagree

28) Making decisions is a problem for your family

◯ Strongly Agree ◯ Agree ◯ Disagree ◯ Strongly Disagree

29) You are able to make decisions about how to solve problems

◯ Strongly Agree ◯ Agree ◯ Disagree ◯ Strongly Disagree

30) You don't get along well

◯ Strongly Agree ◯ Agree ◯ Disagree ◯ Strongly Disagree

31) You confide in each other

◯ Strongly Agree ◯ Agree ◯ Disagree ◯ Strongly Disagree

We want to know more about what you think, how you feel, and what you do. Please select how true each sentence is for you.

		Not at all True	A little True	Pretty True	True	Very True
32)	Your life won't be good until you feel happy.	○	○	○	○	○
33)	Your thoughts and feelings mess up your life.	○	○	○	○	○
34)	If you feel sad or afraid, then something must be wrong with you.	○	○	○	○	○
35)	The bad things you think about yourself must be true.	○	○	○	○	○
36)	You don't try out new things if you're afraid of messing up.	○	○	○	○	○
37)	You must get rid of your worries and fears so you can have a good life.	○	○	○	○	○
38)	You do all you can to make sure you don't look dumb in front of other people.	○	○	○	○	○
39)	You try hard to erase hurtful memories from your mind.	○	○	○	○	○
40)	You can't stand to feel pain or hurt in your body.	○	○	○	○	○
41)	If your heart beats fast, there must be something wrong with you.	○	○	○	○	○
42)	You push away thoughts and feelings that you don't like.	○	○	○	○	○
43)	You stop doing things that are important to you whenever you feel bad.	○	○	○	○	○
44)	You do worse in school when you have thoughts that make you feel sad.	○	○	○	○	○
45)	You say things to make you sound cool.	○	○	○	○	○
46)	You wish you could wave a magic wand to make all your sadness go away.	○	○	○	○	○
47)	You are afraid of your feelings.	○	○	○	○	○
48)						

You can't be a good friend when you feel upset.	○	○	○	○	○

Please select the word that shows how often each of these things happens to you. There are no right or wrong answers.

		Never	Sometimes	Often	Always
49)	You feel sad or empty.	○	○	○	○
50)	You worry when you think you have done poorly at something	○	○	○	○
51)	You feel afraid of being on your own at home	○	○	○	○
52)	Nothing is much fun anymore	○	○	○	○
53)	You worry that something awful will happen to someone in your family	○	○	○	○
54)	You are afraid of being in crowded places (like shopping centers, the movies, buses, busy playgrounds)	○	○	○	○
55)	You worry what other people think of you	○	○	○	○
56)	You have trouble sleeping	○	○	○	○
57)	You feel scared to sleep on your own	○	○	○	○
58)	You have problems with my appetite	○	○	○	○
59)	You suddenly become dizzy or faint when there is no reason for this	○	○	○	○
60)	You have to do some things over and over again (like washing hands, cleaning or putting things in a certain order)	○	○	○	○
61)	You have no energy for things	○	○	○	○
62)	You suddenly start to tremble or shake when there is no reason for this	○	○	○	○
63)	You cannot think clearly	○	○	○	○
64)	You feel worthless	○	○	○	○
65)	You have to think of special thoughts (like numbers or words) to keep bad things from happening	○	○	○	○
66)					

	You think about death	◯	◯	◯	◯
67)	You feel like you don't want to move	◯	◯	◯	◯
68)	You worry that you will suddenly get a scared feeling when there is nothing to be afraid of	◯	◯	◯	◯
69)	You are tired a lot	◯	◯	◯	◯
70)	You feel afraid that I will make a fool of myself in front of people	◯	◯	◯	◯
71)	You have to do some things in just the right way to stop bad things from happening	◯	◯	◯	◯
72)	You feel restless	◯	◯	◯	◯
73)	You worry that something bad will happen to you	◯	◯	◯	◯

Please check each item showing how frequently these comments were true for you during the past 7 days.

		Not at all	Rarely	Sometimes	Often
74)	Do you think about it even when you don't mean to?	◯	◯	◯	◯
75)	Do you try to remove it from your memory?	◯	◯	◯	◯
76)	Do you have difficulty paying attention or concentrating?	◯	◯	◯	◯
77)	Do you have waves of strong feelings about it?	◯	◯	◯	◯
78)	Do you startle more easily or feel more nervous than you did before it happened?	◯	◯	◯	◯
79)	Do you stay away from reminder of it (e.g. places or situations)?	◯	◯	◯	◯
80)	Do you try not to talk about it?	◯	◯	◯	◯
81)	Do pictures about it pop into your mind?	◯	◯	◯	◯
82)	Do other things keep making you think about it?	◯	◯	◯	◯
83)	Do you try not to think about it?	◯	◯	◯	◯
84)	Do you get easily irritable?	◯	◯	◯	◯
85)	Are you alert and watchful when there no obvious need to be?	◯	◯	◯	◯

86)

Do you have sleep problems?	◯	◯	◯	◯
