# Supplementary material for: Pediatric reporting of genomic results study (PROGRESS): a mixed-methods, longitudinal, observational cohort study protocol to explore disclosure of actionable adult- and pediatric-onset genomic variants to minors and their parents
Source: BMC Pediatr. 2020 May 15;20:222. doi: 10.1186/s12887-020-02070-4 (PMC7227212; doi:10.1186/s12887-020-02070-4)
Supplement: Supplementary file 6 — Additional file 6. Interview guide for semi-structured interviews with a subset of parents of minors (ages 0–17). [file 12887_2020_2070_MOESM6_ESM.pdf]

PROGRESS
Qualitative Interview Guide – Parent

Introduction: Thank you for agreeing to take part in this study. As a reminder, your participation in this study is voluntary. As you recall, we expect this part of the study to take up to an hour. Your responses will help us improve our program and learn more about what it is like to have a child with a MyCode DNA result. I will ask you some broad questions and then follow up with some more specific questions about your experience with receiving genetic information from MyCode. Everything you tell me will be kept confidential. This means that your interview responses will only be shared with research team members. When we write our report on this and the other interviews we are conducting, nothing in our report will identify you. Please be honest with your responses. You can say whatever you want – nothing will hurt my feelings and nothing you say will have a negative effect on your care. We will record this conversation, but the transcript from the conversation will not include any information that identifies you. Please remember: you don't have to talk about anything you don't want to talk about. You can decline to answer any question and you may end the interview at any time.

Is it OK if I start recording now?

<u>Opening question:</u> Please tell me about your experience learning about your child's MyCode result.

Probe: Please tell me more.

Stem	Probes	Constructs
What are your thoughts on the process for finding out about this result?	• What do you remember the genetic counselor saying about the result?<br>• What did you like about this process?<br>• Is there anything you wish were different about the process?<br>• How was the amount of information you discussed with your genetic counselor?<br>• What other resources would you like for the genetics team to provide when reporting results?	Results reporting procedures
What types of things have you done for your child's health since learning the result?	• Any changes in your child's medical care?<br>• Is your child doing anything differently, like diet or exercise, since learning the result?	Child's health behaviors, including lifestyle changes
Have you done anything for your health since learning the result?	• Have you had genetic testing for the DNA result your child has?<br>○ [If have had testing]: How do you feel about your/your partner's role in passing this result to your child?<br>• Any changes to your medical care?<br>• How about changes in your health habits, like diet and exercise?	Parental health behaviors, including cascade testing uptake and lifestyle changes<br>Guilt
Now that you have gotten this result, how do you feel about your decision to have your child join MyCode?	• How satisfied are you with your decision to have your child join MyCode?<br>• Do you have any regrets or concerns related to your decision to have your child join MyCode?	Decisional satisfaction/regret

On the whole, does this result feel like positive or negative information?	• How do you see it helping you or your family?<br>• What negative outcomes do you worry about?<br>○ For example, costs of medical care or insurance?	Perceived benefits<br>Perceived harms<br>Insurance, social, employment, education discrimination
How much has this result been on your mind since you found out about it?	• How have you handled these feelings and thoughts?<br>○ How have your reactions to this result been similar to or different from reactions to other medical information you've learned about your child?<br>○ Have you needed emotional support related to this result?<br>▪ [If no]: Tell me why you think that you've not needed any emotional support?<br>▪ [If yes]: If you felt that need, from whom or from what source did you seek emotional support?<br>• Where are you most likely to turn for emotional support?<br>○ [If response is uninformative]: Some people turn to friends, others to family, and others to the Internet or social media like Facebook – which do you prefer?<br>• To what degree has the result made you worry that any health issues your child has are related to the result?<br>• What effect has this result had on how you think about your child's future?<br>• Have you noticed any change in your relationship with your child since you learned the result?	Anxiety & depression<br>Psychosocial response<br><br>Psychosocial support (parent)<br><br>Vulnerable child syndrome<br>Misattribution of symptoms<br>Life goals, reproductive decisions, restricted life choices<br>Parent-child relationship
Have you made a decision about whether to tell your child about his/her DNA result?	• What were your reasons for that decision?<br>○ [If child is aware]: How has s/he responded to this information?	Family communication (child)<br>Psychosocial support (child)
Which relatives have you told about the result?	• How have they reacted?<br>• Are there any relatives you chose not to tell?<br>○ Why?<br>• Which relatives want to be tested for the DNA result?<br>○ [If able to think of relatives who want testing]: What reasons have they given you for wanting to be tested?<br>○ [If unable to think of relatives who want testing]: What concerns (if any) have your relatives discussed with you about being tested?<br>• Have you noticed any changes in your family interactions since telling your family about the result?<br>• Do you think your family has been acting differently towards your child?	Family response<br><br>Cascade testing intention<br><br>Family relationships

Which friends have you told about this result?	• How have they reacted?<br>• Do you feel that the friends you have told treat your child any differently?	Psychosocial support

Closing: Thanks again for taking the time to talk with me. Your willingness to talk will be very helpful for our study team, and we hope for you, too. [If participant mentioned any topics that merit correction/clarification, say: "I want to mention something that came to mind while we were talking." And then clarify the misconception.] Please call us at XXX-XXX-XXXX if you have any questions about this study or about your DNA result. We will call you in about 6 months to do a survey that will update us on how things are going with your MyCode DNA result.
